# Machine Learning Integration of Eye-Tracking and Cognitive Screening for Detecting Cognitive Impairment

**DOI:** 10.3390/jemr19030057

**Published:** 2026-05-20

**Authors:** Joan Goset, Clara Mestre, Valldeflors Vinuela-Navarro, Mikel Aldaba, Mar Ariza, Neus Cano, Bàrbara Delàs, Olga Gelonch, Maite Garolera, REHAB Project Collaborative Group, Meritxell Vilaseca

**Affiliations:** 1 Center for Sensors, Instruments and Systems Development, Universitat Politècnica de Catalunya-BarcelonaTech, 08222 Terrassa, Spain; joan.goset@upc.edu (J.G.); valldeflors.vinuela@upc.edu (V.V.-N.); mikel.aldaba@upc.edu (M.A.); meritxell.vilaseca@upc.edu (M.V.); 2 Departament de Psicologia Clínica i Psicobiologia, Universitat de Barcelona, 08035 Barcelona, Spain; mariza@ub.edu; 3 Clinical Research Group for Brain, Cognition and Behavior, Consorci Sanitari de Terrassa (CST), 08227 Terrassa, Spain; ncanom@cst.cat (N.C.); ogelonch@cst.cat (O.G.); mgarolera@cst.cat (M.G.); 4 Department of Psychology, Faculty of Medicine and Health Sciences, Universitat Internacional de Catalunya, 08195 Sant Cugat del Vallès, Spain; 5 Ophthalmology Service, Consorci Sanitari de Terrassa (CST), 08227 Terrassa, Spain; bdelas@cst.cat; 6 Neuropsychology Unit, Hospital de Terrassa, Consorci Sanitari de Terrassa (CST), 08227 Terrassa, Spain

**Keywords:** eye tracking, post-COVID-19 condition, cognitive impairment, machine learning, eye movements, neuropsychological tests

## Abstract

Cognitive impairment is common in Post-COVID-19 Condition (PCC), yet full neuropsychological testing remains resource-intensive. Because eye movements are known to be altered in certain cognitive disorders, Eye-Tracking (ET) offers a fast, non-invasive complementary approach for large-scale screening. This study aimed to predict neuropsychological test scores of participants with PCC from ET metrics using machine and deep learning models. ET data was collected from 172 participants performing a battery of visual tasks designed to elicit smooth pursuit and fixational eye movements, as well as pupil responses to light. Cognitive performance was assessed through established neuropsychological tests. We applied regression and classification models (e.g., Random Forest, XGBoost, and deep neural networks) to predict neuropsychological performance. Models were trained using ET data alone and in combination with the Montreal Cognitive Assessment (MoCA) scores, a widely used neuropsychological test for global cognitive screening. Although predicting individual test scores was challenging, combining them into a global composite measure improved performance. Model sensitivity and specificity reached 88% and 34% using ET data alone, and 87% and 60% when integrating ET with MoCA. This last trained model outperformed the conventional MoCA, highlighting the potential of ET as a rapid screening support tool for cognitive assessment.

## 1. Introduction

Post-COVID-19 condition (PCC) describes symptoms that persist after SARS-CoV-2 infection, typically emerging about three months after the acute illness and lasting for at least two months, without being explained by another medical condition [1]. Symptoms may represent a continuation of the acute episode or arise only after apparent recovery [1]. Recent studies suggest that PCC affects roughly 43% of individuals following infection [2], with higher rates observed in patients who experienced severe acute disease requiring hospitalization [2]. Many of these patients experience a broad range of complaints affecting multiple organ systems. Reported manifestations include fatigue, headache, loss or distortion of smell and taste, and dyspnea [3].

Subjective cognitive complaints are also common, with patients describing “brain fog”, a non-specific term referring to reduced mental clarity and slowed thinking, concentration difficulties, and episodes of confusion. Neuropsychological testing has revealed cognitive impairments (i.e., objective reductions in cognitive performance relative to normative expectations) in several domains, such as executive functioning, working memory, and processing speed, following PCC [4,5,6,7]. These complaints are strongly associated with fatigue and depressive symptoms, and show a weak relationship with objective cognitive performance measured with neuropsychological testing. This suggests that cognitive impairment may be present even in patients without cognitive complaints, regardless of hospitalization status [4]. Cognitive impairment is often mild and heterogeneous, varying across individuals in the domains affected, severity, and the co-occurring symptoms contributing to the impairment. Altogether, distinguishing and predicting cognitive impairment remains challenging, particularly at the individual level.

Neuropsychological testing is the established reference for the assessment of cognitive impairment across neurological and psychiatric conditions. In PCC, as in other contexts, neuropsychological test batteries are used to quantify cognitive performance and identify domain-specific impairments. However, their application in large-scale or repeated assessments outside specialized neuropsychological settings is limited by practical factors, including long administration times, the need for trained professionals, and susceptibility to fatigue, education, and mood among others. Brief cognitive screening tools, such as the Montreal Cognitive Assessment (MoCA), were developed to address some of these limitations, offering a faster and more accessible alternative. While the MoCA is commonly adopted due to its sensitivity to mild cognitive impairment [8,9], its performance is influenced by demographic factors such as education level and age, which motivated the introduction of score adjustments [10]. Subsequent studies and meta-analyses have shown that optimal MoCA cut-off scores vary across populations and clinical settings, and that cross-cultural adaptations exhibit variability in sensitivity and specificity [10,11,12]. This MoCA variability is particularly relevant when the cognitive impairment assessed is subtle and heterogeneous, as observed in PCC. From a practical perspective, the MoCA must be administered by trained and certified professionals to ensure standardized delivery and scoring [8]. Taken together, these limitations motivate interest in complementary, objective screening approaches.

One promising approach involves the use of objective, measurable motor and physiological responses as indirect predictors of cognitive performance. Eye-Tracking (ET) has been increasingly used as an objective, non-diagnostic, non-invasive, and time-efficient method for characterizing oculomotor behavior associated with cognitive impairment [13,14]. Demonstrating that ET metrics can reliably predict neuropsychological performance at the individual level would support their use as a complementary tool to instruments like the MoCA. Numerous studies have shown that specific ET metrics relate to distinct cognitive domains [15]. This association between ET data and cognition is rooted in the fact that eye movements are controlled by a complex oculomotor system involving cortical and subcortical brain regions [16,17,18].

Measures obtained during smooth-pursuit eye movements have been found to identify individuals with neurological or neurodegenerative conditions [19,20]. Performing smooth pursuit tasks activates multiple brain areas, including the fronto-insular cortex, anterior cingulate cortex, superior colliculi, supplementary motor area, and thalamus [15]. The engagement of these regions suggests that smooth pursuit performance depends on neural systems overlapping with executive functions such as attention and response control [15]. Consistent with this distributed network involvement, smooth pursuit deficits are observed across multiple disorders: Alzheimer’s and Parkinson’s diseases are characterized by reduced velocity gain and frequent saccadic intrusions that worsen with disease progression [21,22]; multiple sclerosis patients exhibit low-gain pursuit associated with lesions affecting frontal eye field, cerebellar, and vestibular pathways [23]; amyotrophic lateral sclerosis patients were found to have impaired pursuit eye movement which was suggested as a sign of extrapyramidal or supratentorial pyramidal involvement [15]. Smooth pursuit impairment is described as “trait characteristic” in schizophrenia, appearing to have a genetic component [24]. Finally, patients with PCC showed significantly higher amplitude of saccades during smooth pursuit compared to healthy controls [25].

Fixation duration and stability reflect the brain’s ability to sustain attention and maintain visual engagement [15,26,27]. Rather than being a passive absence of movement, fixation is an active process that requires continuous attention and suppression of reflexive saccades [28]. Neuroimaging studies indicate that frontal and cingulate regions (bilateral dorsolateral prefrontal cortex, anterior cingulate cortex, and frontal eye fields) contribute to control mechanisms that stabilize gaze [15,28]. When these mechanisms are compromised, characteristic patterns emerge: mild cognitive impairment patients tend to show shorter average fixation durations, likely reflecting impaired sustained attention [26], while Alzheimer’s disease is associated with large “intrusive saccades” during fixation, indicative of a failure in cortical suppression [21,28]. Similarly, increased fixation instability has been reported in PCC patients relative to healthy controls [25].

Pupillary behavior is often assessed using metrics such as latency to pupil constriction, peak pupil constriction, and baseline pupil diameter [17]. Beyond reflecting light reflexes, task-evoked pupillary responses are sensitive to changes in arousal, attentional control, and mental effort [17]. Whereas pupillary responses are less commonly assessed than fixations, they offer accurate information about executive function and are considered potential biomarkers for evaluating conditions like Alzheimer’s disease and Parkinson’s disease [15,16,26]. Additionally, recent research found that PCC patients showed significantly reduced pupil responses to light stimulation (both constriction and dilation) compared to a control group [25]. Together, these findings suggest that ET provides rich, multivariate behavioral data that may be sensitive to cognitive impairment not readily captured by conventional screening testing.

However, because eye movements and pupil response metrics reflect the output of multiple control systems, the cognitive information captured by ET is distributed across numerous interrelated parameters. No single metric is sufficient to characterize cognitive impairment; rather, it is the combination and interaction of multiple measures that best captures cognitive performance. This complexity makes ET data well-suited for machine learning approaches aimed at predicting cognitive performance, which can integrate multiple parameters simultaneously and capture nonlinear relationships. Machine learning methods integrated with ET metrics have been used before to differentiate individuals with PCC from healthy controls [29]. More broadly, computational approaches to ET analysis have been applied across neurological and psychiatric populations supporting the feasibility of using machine learning to extract clinically meaningful information from oculomotor data [16,30,31].

In previous work, we reported that individuals with PCC exhibit specific ET parameters (from fixation, smooth pursuit and pupil dynamics) associated with neuropsychological outcomes [32]. Data-driven analyses, including principal component analysis and clustering, further indicated that ET features can differentiate patient subgroups with distinct cognitive profiles. While these findings provided evidence that ET can capture clinically relevant information at the group level, they did not address whether ET could support prediction of cognitive performance at the individual level.

Given the evidence linking eye movements and pupil responses with cognitive performance, this study aims to examine whether ET data combined with machine learning can predict performance on neuropsychological tests. The study has two objectives: first, to evaluate whether ET parameters can predict neuropsychological test scores; and second, to assess whether ET features can distinguish between altered and non-altered cognitive performance as determined by neuropsychological assessment.

## 2. Materials and Methods

### 2.1. Participants

This study included two samples: a primary cohort that completed both ET and neuropsychological assessments, and an independent normative reference sample used to establish baseline cognitive performance. Individuals with PCC and healthy participants who were enrolled in the Nautilus clinical study (ClinicalTrials.gov Identifier: NCT05307575) and in the Rehab COVID project (ClinicalTrials.gov Identifier: NCT05846126) were invited to take part in this study. Participants were recruited through the Consorci Sanitari de Terrassa (Terrassa, Barcelona, Spain) from November 2021 to October 2024. All procedures were conducted in accordance with the Declaration of Helsinki and received approval from the Drug Research Ethics Committee (CEIm) of the Consorci Sanitari de Terrassa (reference: 02-20-107-070).

Participants with PCC were eligible for inclusion if they met the following criteria: (1) A confirmed diagnosis of COVID-19 based on WHO guidelines, with signs and symptoms present during the acute phase; (2) a minimum of 12 weeks since the acute infection; (3) persistence of post-infection symptoms consistent with PCC; (4) age between 18 and 65 years.

All participants in the ET study were excluded if they presented any of the following: (1) A prior diagnosis of psychiatric, neurological, neurodevelopmental, or systemic conditions known to affect cognition; (2) motor or sensory impairments that could interfere with neuropsychological evaluation; (3) type 1 or type 2 diabetes; (4) history of intraocular or refractive surgery; (5) glaucoma or any retinal pathology; (6) diagnosed or suspected strabismus; (7) stereopsis poorer than 100 arcseconds; (8) binocular near visual acuity worse than 0.2 logMAR or binocular visual acuity worse than 0.15 logMAR (both corrected); (9) participants with high myopia, defined as a refractive error greater than −6 diopters. A total of 191 participants completed the ET protocol. After applying exclusion criteria, 172 participants were retained for the primary ET-neuropsychological analyses (mean age = 49.69 ± 7.58 years; 79% female).

A normative reference sample was recruited to establish baseline cognitive performance for the neuropsychological assessments. It comprised 133 participants, who completed neuropsychological testing only. For this normative sample, exclusion criteria related to prior psychiatric, neurological, neurodevelopmental, or systemic conditions affecting cognition, as well as motor or sensory impairments (exclusion criteria 1 and 2 above), were applied. The normative reference sample was used to derive demographic-adjusted z-scores for the neuropsychological test outcomes.

### 2.2. Neuropsychological Tests

All neuropsychological assessments were administered individually by trained clinical neuropsychologists in a fixed order designed to minimize fatigue and interference between tasks. More cognitively demanding tasks, such as those involving executive function and working memory, were scheduled earlier in the session when mental load was lower. Short breaks were permitted when needed. Since no single task can fully capture a cognitive domain, multiple complementary tests were used together as indicators of each domain.

The MoCA is a brief, cognitive screening tool developed to improve the detection of Mild Cognitive Impairment (MCI). It is designed to be administered in approximately 10 min and covers a broad set of cognitive domains. It consists of short-term memory assessment through two learning trials of five words followed by delayed recall; visuospatial abilities assessed using clock drawing and three-dimensional cube copying; executive functioning evaluated through a Trail Making-type alternation task, phonemic verbal fluency, and verbal abstraction tasks; attention and working memory measured by repeating digits forward and backward, target-detection tapping, and serial subtraction tasks; language assessment through confrontation naming of low-familiarity animals and repetition of complex sentences; and orientation testing for time and place. Its validation results indicated higher sensitivity for detecting MCI than other common screening tests and retaining good specificity [8]. In the context of post-COVID condition, previous work from our group supports the usefulness of including the MoCA as part of brief cognitive screening protocols. In that study, a model including MoCA performance, Digit Symbol, and phonetic verbal fluency contributed to distinguishing individuals with post-COVID condition from healthy controls, showing good sensitivity and acceptable overall discrimination, although specificity was low [4]. Nevertheless, the MoCA remains a screening tool. Its brief multidomain format limits domain-specific interpretation, and it should not be considered a standalone diagnostic instrument or a substitute for comprehensive neuropsychological assessment.

The Digit Symbol test from the Wechsler Adult Intelligence Scale-Third Edition (WAIS-III) was used to assess processing speed and sustained attention. Participants completed a timed task requiring rapid matching of digits to their corresponding symbols. The score reflects the total matches completed [33].

The Digit Span was used to assess attention and working memory. The Digit Span Forward task evaluates attention, requiring participants to repeat sequences of digits in the same order as presented, with span length increasing until performance failed. In contrast, the Digit Span Backward assesses working memory and executive functioning; in this task, participants repeated sequences of digits in reverse order. For both tasks, the score corresponds to the total number of correctly reproduced sequences [33].

The Trail Making Test (TMT) includes two parts. TMT-A evaluates processing speed and attention through a numbered sequencing task, while TMT-B adds an executive component by requiring alternating number-letter sequences. Performance on both parts is scored as the time required to complete the sequence, with longer times indicating poorer performance [34,35,36].

The Stroop Color-Word Test (SWCT) comprises three conditions. In the Word and Color conditions, participants read color words or name color patches, providing measures of processing speed. In the Color-Word condition, they must name the ink color of incongruent color words, which places additional demands on executive-function inhibition. Responses are scored as the number of correct items produced within a fixed time interval [37].

Verbal fluency was assessed through both phonological and semantic tasks. Phonemic fluency was measured using the Controlled Oral Word Association Test (COWAT) with the letters P, M, and R, while semantic fluency was evaluated through an animal-naming task. In both tasks, performance was scored as the number of valid, non-repeated responses produced within one minute [38,39].

### 2.3. Eye Movement Recording

ET recording was conducted in a separate visit from the neuropsychological testing. Participants were screened to confirm that their near visual acuity and binocularity met the study’s inclusion criteria. Participants performed the visual tasks under binocular viewing using a desktop-mounted EyeLink 1000 Plus eye tracker (SR-Research Ltd., Ottawa, ON, Canada) sampling at 1000 Hz. The standard 9-point calibration was performed prior to data acquisition, achieving a mean accuracy of 0.88° ± 0.59°. Eye movements were recorded binocularly. All recordings were conducted under dim illumination to minimize visual distractions.

Participants were seated and their head was supported by a chin rest at 60 cm from an LCD computer screen (1280 × 1024 pixels, 60 Hz, 17″). When needed, participants wore their habitual glasses or contact lenses to correct refractive error. Stimulus presentation followed the configuration used in our previous study [25] and included tasks designed to elicit smooth pursuit and steady fixation. Pupil myosis and mydriasis in reaction to light was also recorded. Smooth pursuit was assessed by asking participants to visually track a small moving dot (0.3°) along horizontal, vertical, and sinusoidal trajectories. During the fixation task, participants fixated a 0.5° central cross. After 10 s, brief peripheral stimuli (distractors) appeared for 30 s, while participants were instructed to maintain fixation on the central target. Pupil responses to light were also recorded using an LED light stimulus. During this procedure, participants fixated a central white cross on a dark background; after a brief interval, the LED light source was illuminated for 5 s and then switched off. Pupil diameter was measured both before and after light onset to quantify pupillary constriction and dilation. All visual stimuli and task sequences were generated and presented using MATLAB v8.5.0.197613, R2015a (MathWorks, Natick, MA, USA) with the Psychophysics Toolbox extension [40,41].

### 2.4. Data Analysis

ET recordings were processed using a combination of the manufacturer’s EyeLink Host Software v5.04 and custom Python v3.8.10 scripts. For the analysis, data from the right eye was used across participants. The native software automatically marked blink periods, and signal segments spanning 200 ms before and after each blink were discarded. It also applied two built-in heuristic filters to attenuate measurement noise. Additional processing steps were performed offline with custom python scripts. Velocity profiles were smoothed using a 21-ms Savitzky–Golay filter (second order), and saccades were detected through an adaptive velocity-based threshold that adjusted to the noise of each recording [42]. Fixation periods were derived after removing all velocity-defined saccades.

A preliminary screening analysis was conducted to identify the ET parameters most strongly associated with neuropsychological performance. Partial Spearman correlations were computed between each ET metric and the neuropsychological test scores, controlling for age and years of education. The complete set of extracted eye movements and pupil-response metrics tested for correlations is available in Supplementary Table S1. Only parameters showing statistically significant associations were retained for modeling. This procedure yielded four features: fixation Root Mean Square error (RMS) with distractors, smooth-pursuit RMS in sinusoidal and vertical trajectories, and pupil area reduction. This analysis showed correlation coefficients in the range of 0.210–0.290 (*p* < 0.05) [32].

For both fixation and smooth pursuit tasks, spatial error at each time sample *i* was defined as the Euclidean distance between gaze position ($x_{{gaze}_{i}}$, $y_{{gaze}_{i}}$) and target position ($x_{{stim}_{i}}$, $y_{{stim}_{i}}$). RMS was computed across the full duration of the trial as in Equation (1):

(1) $$RMS=\sqrt{\frac{1}{N}\sum_{i=1}^{N}{\left(x_{{gaze}_{i}}-x_{{stim}_{i}}\right)}^{2}+{\left(y_{{gaze}_{i}}-y_{{stim}_{i}}\right)}^{2}}$$

where N denotes the total number of gaze samples in the trial.

In the fixation task, the target was static at the central fixation position (0,0), such that $x_{{stim}_{i}}=0$ and $y_{{stim}_{i}}=0$ for all *i*. In the smooth-pursuit task, target coordinates corresponded to the stimulus position at the gaze timestamp. Lower RMS values indicate reduced spatial error, reflecting greater fixation stability or tracking precision. For each participant, RMS was computed across each trial, yielding a single scalar value per condition (fixation with distractors, sinusoidal pursuit, and vertical pursuit).

Finally, pupil area reduction was computed relative to baseline. Baseline pupil area (B) was defined as the mean pupil area during the 1 s interval preceding LED onset ($t_{LED}$):

(2) $$B=\frac{1}{N_{baseline}}\sum_{t=t_{LED}-1\;s}^{t=t_{LED}}A\left(t\right)$$

where $A\left(t\right)$ denotes pupil area at time *t* and $N_{baseline}$ the number of gaze samples in the time interval.

The constriction level (C) was defined as the mean pupil area during the 1 s interval beginning at the first detected local minimum $t=t_{min}$ following LED onset. The detected local minimum must have represented at least a 10% decrease from baseline. $N_{miosis}$ correspond to the number of samples in the time interval.

(3) $$C=\frac{1}{N_{miosis}}\sum_{t=t_{min}}^{t=t_{min}+1\;s}A\left(t\right)$$

Pupil reduction was expressed as the relative percentage decrease from baseline to minimum pupil area, and this value was used in the modeling analyses as presented in Equation (4):

(4) $$\%\;Reduction=\frac{B-C}{B}$$

The proportion of missing data varied across features, with fixation RMS (distractors) showing 2.9% missing values, smooth-pursuit RMS in the sinusoidal condition 4.65%, smooth-pursuit RMS in the vertical condition 4.65%, and pupil are reduction 14.53%.

### 2.5. Machine Learning Models

The first step of the machine learning pipeline involved preparing the dataset, particularly addressing missing values. In our dataset, missing data arose mainly from technical limitations of the ET (e.g., loss of pupil signal due to eyelid closure or poor tracking quality). These cases were handled using multiple imputation with scikit-learn’s IterativeImputer [43], which preserves relationships among variables. Following preprocessing, a range of supervised machine-learning models was then evaluated to predict neuropsychological performance from ET features and age as inputs. Two families of models were considered: regression algorithms, used to estimate each neuropsychological raw test score, and classification algorithms, used to determine whether neuropsychological test performance was altered or not. All models shared a common input feature set comprising four scalar ET measures (fixation RMS error during the distractor condition, smooth-pursuit RMS error along the sinusoidal trajectory, smooth-pursuit RMS error along the vertical trajectory, and pupil area reduction) each computed as a single value per participant and visual task. Participant age was additionally included as a predictor in all models. The dataset was structured in table format, where each row represented one participant and each column corresponded to one predictor variable. Outcome variables, either continuous neuropsychological test scores (for regression) or binary impairment labels (for classification), were handled separately depending on the modeling task. The following subsections describe the specific models and their implementation.

#### 2.5.1. Regression Approach

Five regression approaches were evaluated: (1) Ordinary Least Squares (OLS), (2) Stochastic Gradient Descent (SGD), (3) Extreme Gradient Boosting (XGBoost), (4) Random Forest, and (5) feedforward neural networks. Implementations used scikit-learn, XGBoost for gradient boosting, and TensorFlow/Keras v2.13.0 [44] for neural networks.

Hyperparameter optimization was performed for all machine learning models using Bayesian optimization implemented in the Optuna framework [45] by minimizing the average error across all neuropsychological outcomes. The search spaces evaluated 80 configurations per model. For SGD, the search covered the loss function, penalty type, and regularization strength. For the ensemble tree methods (XGBoost and Random Forest), it included tree depth, number of estimators, subsampling fractions, and regularization parameters. For the feedforward neural network, the search spanned network depth, hidden layer sizes, activation function (ReLU or ELU), dropout rate, L2 regularization strength, learning rate, batch size, and early stopping patience. The full set of optimized hyperparameter values are listed in Supplementary Table S2.

Model performance was evaluated using 5-fold cross-validation (KFold, shuffle = True). In each iteration, models were trained on four folds and evaluated on the held-out fold, ensuring that no participant data used in the training phase participated in the performance evaluation. Out-of-fold predictions (i.e., predictions obtained for each observation when it appears in the test fold) were aggregated across all folds. Predictive performance was assessed using the coefficient of determination (R^2^) and the Root Mean Squared Percentage Error (RMSPE), computed on the pooled out-of-fold predictions.

#### 2.5.2. Classification Approach

The neuropsychological tests battery defined the cognitive outcomes predicted by the models. For each test, outcomes were defined as a binary classification (“altered” vs. “not altered”). Machine learning models were trained using the ET features to predict these classifications. The MoCA was evaluated separately as a screening test and, in some models, was included as an additional predictor.

Because neuropsychological performance varies as a function of demographic factors, classification labels were derived from demographic-adjusted z-scores computed using the normative reference sample (*n* = 133). This sample was used to establish baseline performance by estimating expected scores using an OLS regression model with age, years of education, and gender as predictors. Standardized z-scores were then calculated as deviations from expected performance, as defined in Equation (5), and classified as altered when |z| > 1 (with direction reversed for time-based tests such as TMT) [46].

(5) $$z_{score}=\frac{{Score}_{observed}-{Score}_{expected\;from\;OLS\;regression}}{\sigma_{normative\;sample}}$$

Thus, classification was based on demographically adjusted performance rather than raw test scores or direct group comparisons. For the MoCA, the altered label was defined using the conventional clinical cutoff (score < 26 points).

Although individual tests involve overlapping cognitive processes, neuropsychological performance is commonly interpreted according to the predominant cognitive function required by each task. Accordingly, tests were grouped a priori into cognitive domains reflecting their principal cognitive demand to enable domain interpretation and reduce test variability. Separate composites were created to distinguish between executive function tests that vary on processing speed, and between processing speed tests that vary in their attentional demands. The following composites were examined: Attention (Digit Span Forward and TMT-A), Executive Function 1 (Digit Span Backward, TMT-B-A ratio, Stroop Color-Word, and phonological fluency), Executive Function 2 (Digit Span Backward, TMT-B, Stroop Color-Word, and phonological fluency), Processing Speed 1 (Digit Symbol, TMT-A, Stroop Word, and Stroop Color), and Processing Speed 2 (Digit Symbol, Stroop Word, and Stroop Color). Additionally, a derived global composite was also created. For each composite, the altered state was defined by the presence of two or more altered tests within the composite, while the global composite was defined by considering all neuropsychological tests together (excluding MoCA). The global composite should be interpreted as an aggregate indicator of overall cognitive performance within the study, rather than as a clinical diagnosis. It reflects the general principle of multi-domain impairment described in Petersen’s MCI framework [47] without implying a clinical MCI classification.

Two families of classifiers were evaluated: Linear Discriminant Analysis (LDA) and Random Forest classification (RF). Models were implemented using scikit-learn. Independent hyperparameter optimization was conducted for each neuropsychological outcome to ensure the maximum performance. This process utilized the Optuna framework [45] for Bayesian optimization, targeting the maximization of the Area Under the Receiver Operating Characteristic Curve (ROC-AUC). Final optimized hyperparameters are presented in Supplementary Table S3. The ROC-AUC was used as the primary metric for model optimization and comparison because it provides a threshold-independent evaluation of classifier discrimination. A 5-fold cross-validation procedure was applied following the same strategy ensuring that no participant data used in the training phase participated in the performance evaluation. Out-of-fold predictions were aggregated across folds, and performance metrics were computed on the pooled predictions. Final comparisons between LDA and Random Forest models were based on these out-of-fold predictions, with McNemar’s test used to assess statistical differences in classification performance.

MoCA was evaluated as a standalone classifier to serve as a clinical screening reference for comparison with ET-based models. The conventional cut-off score of <26 was applied. The MoCA was selected because it is a brief and widely used screening instrument that provides a global measure of cognitive functioning across multiple domains, with minimal administration time. Because lower MoCA scores indicate greater cognitive impairment, scores were inverted during ROC computation.

Additionally, motivated by the potential of complementary classification combining ET and MoCA, a model was trained using the MoCA scores, besides the ET features and age, as inputs to predict the abovementioned binary classification. The weight of the MoCA feature was systematically varied by duplicating it within the feature pool, thereby increasing its probability of selection during tree construction [48]. Multiple models with different MoCA weights were trained and evaluated using the same stratified 5-fold cross-validation procedure described above. Classification models’ performance was assessed as described in the previous section.

Given the relevance of the global composite as an indicator of multi-test cognitive alteration aligned with screening applications, a final comparison focused on the global composite outcome. Therefore, ROC curves were computed for the global composite to compare the classification performance of the evaluated models. For the RF-based models, confusion matrices were derived using thresholds selected to ensure a minimum sensitivity of 0.85, reflecting a screening-oriented criterion. Differences in model performance were assessed for statistical significance using McNemar’s test.

To evaluate the relative contribution of each predictor to the model’s decisions, a permutation feature importance analysis was performed on the ET and MoCA combined model. This technique quantifies importance by measuring the mean decrease in the Area Under the Receiver Operating Characteristic Curve (ROC-AUC) when the values of a specific feature are randomly permuted. A total of 50 permutations were performed for each feature.

## 3. Results

### 3.1. Neuropsychological Test Scores

Table 1 presents descriptive statistics for all neuropsychological raw test scores in the primary cohort that completed both the ET and neuropsychological protocols (*n* = 172). For the majority of the neuropsychological tests in our battery, higher raw scores indicate superior cognitive performance. For the TMTs, where the score represents the completion time in seconds, lower scores reflect better performance.

### 3.2. Regression Results

Table 2 shows the performance results of the five different regression algorithms to predict continuous neuropsychological test scores from ET features and age.

Across all neuropsychological tests and models, R^2^ values were mostly close to zero and frequently negative, indicating that none of the models outperformed a baseline predicting the mean score. Corresponding RMSPE values ranged from 11.95% to 105.16% across tests and models, indicating large differences between predicted and actual scores.

### 3.3. Classification Results

Table 3 presents the classification performance of LDA and RF using only ET features and age as inputs (LDA-ET, RF-ET), the MoCA and a model-level integration incorporating the MoCA score besides the ET data and age as inputs to the RF (RF-ET + MoCA). Across individual neuropsychological tests and composites, the table reports the proportion of participants classified as altered and the ROC-AUC value of each classification approach. For the RF-ET + MoCA model, optimal performance was achieved when MoCA and ET features were equally weighted.

LDA-ET and RF-ET showed moderate discriminative performance for the different tests and composites, with AUC values ranging between 0.48 and 0.72. Since no significant differences were found between them across all tests (McNemar’s test, all *p* > 0.07), the RF-ET classifier was retained for subsequent analyses due to its greater flexibility in capturing nonlinear relationships. The MoCA alone yielded the higher AUC values for several outcomes ranging from 0.50 to 0.75. The combined RF-ET + MoCA model showed the highest AUC values for most outcomes, including the global composite (AUC of 0.80).

**Figure 1:**
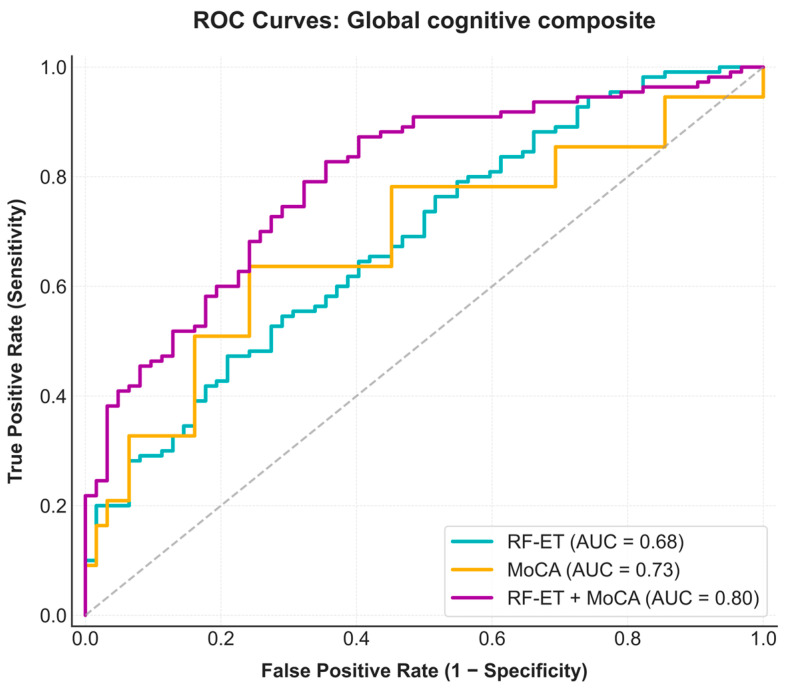
Receiver operating characteristic (ROC) curves for prediction of the global composite. Three models are shown: ET features alone using a RF-ET, the MoCA used as a standalone screening measure, and the RF-ET + MoCA. The combined RF-ET + MoCA model achieved the highest discriminative performance (AUC = 0.80), compared with MoCA alone (AUC = 0.73) and the ET model alone (AUC = 0.68). The dashed diagonal line indicates chance-level performance (AUC = 0.50).

**Figure 2:**
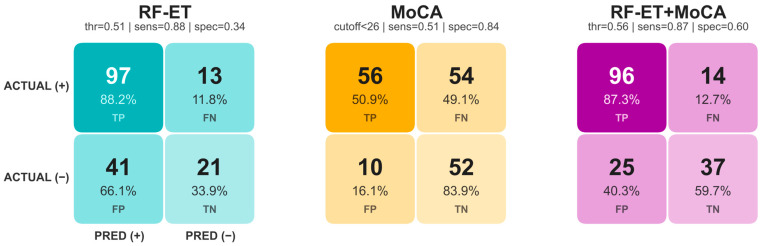
Confusion matrices for the global composite classification using the RF-ET, the MoCA as a standalone classifier, and the combined RF-ET + MoCA. Values represent classification counts, with percentages. Percentages are calculated relative to the total number of cases within each actual class (row-normalized). Classification thresholds (thr) and cut-off for MoCA, sensitivity (sens) and specificity (spec) of each model are presented above the confusion matrices.

The classification performance of three approaches for the global composite was further examined using ROC curves (Figure 1), confusion matrices (Figure 2), precision-recall curves (Supplementary Figure S1), and several performance metrics (Supplementary Figure S2). For the confusion matrices, classification thresholds were selected from the ROC curves by prioritizing sensitivity ≥ 0.85. For the MoCA, the confusion matrix was derived using the conventional clinical cutoff (<26 indicating altered performance).

No significant differences were observed between the RF-ET and MoCA classifiers (χ^2^ = 0.92, *p* = 0.34). The RF-ET + MoCA model showed improved performance compared with RF-ET alone (χ^2^ = 4.00, *p* = 0.046) and with MoCA alone (χ^2^ = 10.11, *p* = 0.001).

**Figure 3:**
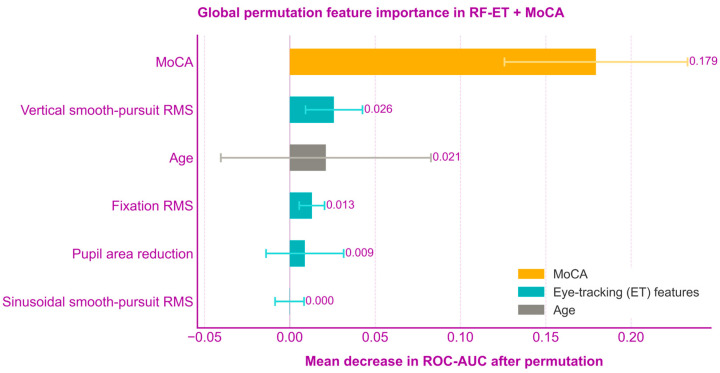
Global permutation feature importance for the multimodal RF-ET + MoCA classification model. Importance is quantified as the mean decrease in the Area Under the Receiver Operating Characteristic Curve (ROC-AUC) after randomly permuting each feature’s values. The MoCA score emerged as the most influential predictor for the global cognitive composite, followed by Vertical smooth-pursuit RMS and Age. Error bars represent the standard deviation across 50 permutations, reflecting the stability of each feature’s contribution to the model’s performance.

Permutation feature importance analysis (Figure 3) was subsequently performed to examine the relative contribution of each predictor to the multimodal RF-ET + MoCA classifier. MoCA emerged as the most influential individual predictor, whereas several ET-derived measures, particularly Vertical smooth-pursuit RMS, also contributed positively to the model’s performance.

## 4. Discussion

This study evaluated the ability of ET metrics to predict neuropsychological performance and to distinguish between altered and non-altered cognitive status in PCC, both independently and in combination with the MoCA. The assessment aimed to characterize cognitive performance across key domains rather than to establish a diagnostic determination. The main findings indicate that while ET-based models poorly predicted individual test scores, they provided complementary information that improved classification when combined with the MoCA, especially at the global level. These results suggest that ET metrics may provide additional, objective signals of cognitive impairment that are not fully captured by standard screening tools.

The regression analyses revealed that ET features could not accurately predict individual neuropsychological test raw scores. Across all tests and algorithms, the models performed poorly: R^2^ values fluctuated around 0, meaning they failed to exceed a baseline that predicts the average score for all participants. The RMSPE were correspondingly high, in some cases exceeding 100%, indicating large differences between predicted and actual scores. Non-linear models (XGBoost and RF) showed slightly better performance relative to linear models but remained close to the baseline. Neural networks produced the most negative R^2^ scores. Across models of varying complexity, hyperparameter optimization favored high regularized or shallow architectures, suggesting a weak signal between ET predictors and neuropsychological scores. Altogether, these findings indicate that the selected ET metrics and current task design may not be sufficient for estimating precise scores on individual cognitive tests, as the relationship between these specific predictors and neuropsychological performance appears to be weak and noisy. It is possible that more complex oculomotor parameters or tasks with higher cognitive loads could provide the additional signal required for robust prediction. Beyond the methodological frame, this limited predictive power also likely reflects the clinical complexity of PCC. Cognitive impairment in this population is characteristically subtle and highly heterogeneous. This diffuse presentation, combined with high inter-individual variability in symptoms makes precise score estimation challenging at the individual level [4,5,6,7].

To our knowledge, the use of ET data to predict neuropsychological test scores via regression has not been previously reported. Regression requires accurate estimation of scores and is highly sensitive to prediction error, particularly when cognitive impairments have low signal-to-noise ratio as is the case in PCC. In contrast, classification models reduce test-specific noise and may better capture subtle deviations from normal efficiency.

Based on this rationale, cognitive performance was evaluated using binary classification (altered or non-altered). Using only ET parameters and age as predictors, two classification algorithms were evaluated: LDA and RF. ET-only models showed comparable performance according to the McNemar’s test and AUC values around ~0.53-0.70, indicating modest but consistent discriminative ability. Given the comparable performance between models, RF was selected for subsequent analyses due to its greater capacity to capture non-linear relationships. However, RF has reduced interpretability compared with linear models like LDA because predictions are generated from the aggregation of many decision trees rather than a single equation.

To contextualize the performance of ET classifiers, the MoCA was evaluated as a standalone classifier across the same individual tests and composite cognitive domains. Overall, MoCA outperformed ET-based models, achieving higher AUC values in most outcomes. However, its performance was not uniform across domains, showing limited or below-chance discrimination in tasks such as TMT-A and TMT-B, as well as comparable performance in semantic fluency. These results highlight both the strength of MoCA as a global screening tool and some domain-specific limitations in our population sample.

Given that MoCA and ET metrics capture distinct aspects of cognitive function, multimodal integration was evaluated to combine complementary sources of information. The integrated RF-ET + MoCA model achieved AUC values that exceeded those of the RF-ET classifier alone and were often higher than those of MoCA. Notably, the highest classification performance for the integrated model was observed in the global composite (AUC = 0.80), the Executive Function 2 composite (AUC = 0.81), and the Stroop Color-Word test (AUC = 0.80). These outcomes share a dominance on executive control and task complexity, involving the integration of working memory, inhibition, and cognitive flexibility [33,34,35,36,37,38,39]. While MoCA alone already demonstrated good discriminative performance in these domains, the addition of ET features further improved classification. This pattern suggests that combination of ET and MoCA capture information that enhances the global assessment offered by standard screening measures. Permutation feature importance analysis (Figure 3) showed that MoCA was the strongest individual contributor to the multimodal classifier. This finding is expected, given that MoCA shares conceptual and clinical overlap with the neuropsychological outcomes used for classification. Nevertheless, ET metrics, particularly vertical smooth-pursuit RMS and fixation RMS, also contributed positively to classification performance. Although the contribution of individual ET variables was modest, their combined contribution supported the complementary value of ET biomarkers. Furthermore, the weighted RF architecture was specifically designed to balance feature sampling between MoCA and ET variables during tree construction. Therefore, the predominance of MoCA in the feature importance analysis likely reflects both its strong predictive signal and its weight within this multimodal framework.

ET metrics reflect underlying cognitive control processes. Fixation stability reflects the ability to sustain attention and suppress reflexive saccades, while increased saccadic intrusions may indicate reduced inhibitory control. Similarly, smooth pursuit performance depends on predictive tracking and continuous error correction, processes that rely on the integration of attention and executive control [18,49]. Together, these measures provide indices of cognitive efficiency at the process level, particularly in terms of attention, inhibition, and executive regulation [50]. In contrast, neuropsychological test scores reflect what level of performance was ultimately achieved rather than the underlying processes involved. While these two levels of measurement are related, they are not equivalent. This distinction becomes particularly relevant in PCC, where cognitive impairment is often subtle and may not manifest as clear deficits in neuropsychological test scores. Large cohort studies showed that cognitive impairment in PCC is typically modest in magnitude, heterogeneous across individuals, and diffuse across cognitive domains [4,7]. Further, individual neuropsychological test scores often remain within normative ranges even when individuals report significant subjective cognitive difficulties or demonstrate subtle inefficiencies across multiple domains. This pattern reflects a global reduction in cognitive efficiency rather than focal, severe impairment in a single specific function [4,7].

In this context, detecting a deviation from normative functioning, rather than to estimate exact performance levels, becomes more appropriate for PCC. This aligns with the nature of ET metrics, which capture overall cognitive processing rather than domain-specific performance [50]. Consistent with this perspective, the recent literature has framed cognitive assessment through ET as a classification problem. Prior work emphasized that ET metrics primarily reflect cognitive demands rather than precise performance outcomes [51], while Kim et al. [52] demonstrated this approach by developing a deep learning model that distinguished between normal and impaired executive function during visuospatial memory encoding with high accuracy. Rizzo et al. [53] showed that ET features reliably distinguish cognitive interference states using machine learning classifiers. These studies support the use of ET for screening rather than fine-grained score prediction.

The global composite provides a summary measure of overall cognitive alteration by integrating performance across multiple cognitive domains. It is therefore more aligned with real world screening objectives. This is relevant in PCC, where cognitive impairment tends to be subtle and distributed across domains. The ability of the ET classification models to identify global cognitive status positions itself as a promising complementary screening-level tool. Notably, the ROC profile of the RF-ET model indicates that high sensitivity levels (e.g., ~0.80) can be achieved at moderate specificity (~0.50), an acceptable trade-off in screening contexts where minimizing false negatives is prioritized. This result motivated direct comparison to the MoCA, which has become the most widely used screening tool in both clinical practice and research.

The MoCA was developed and validated for detecting MCI [10] and has since become the most widely used cognitive screening instrument in clinical practice and research. Although PCC-related cognitive impairment differs conceptually from MCI, comparing MoCA performance across these conditions provides a useful framework for understanding its behavior in PCC populations. Consistent with this perspective, large PCC cohort studies have used the MoCA as a global descriptive index, highlighting its role in this population [4,7]. However, the MoCA should still be interpreted as a brief screening instrument rather than a comprehensive diagnostic tool. Its multidomain structure provides an overall estimate of cognitive status but offers limited domain-specific characterization and does not replace a full neuropsychological assessment. The classification performance of the MoCA observed in the present study regarding the global composite differs from that reported in the literature on MCI. A recent systematic review and meta-analysis by Islam et al. [11], largely based on case–control studies applying Petersen criteria for MCI, reported high sensitivity and moderate specificity using the recommended cut-off score of 26. In contrast, we observed lower sensitivity and higher specificity when applying the MoCA within our heterogeneous PCC cohort with the same cut-off value. This discrepancy is likely attributable to differences in study design and population characteristics. Most existing MoCA validation studies compared individuals with cognitive impairment (typically recruited from memory clinics) against healthy controls. This case–control design samples individuals from opposite ends of the cognitive spectrum, potentially inflating sensitivity through spectrum bias. In contrast, subtle cognitive impairments often fail to produce score reductions, leaving many cognitively altered individuals at or near the screening threshold rather than clearly below it. This increases false negatives and reduces sensitivity. Conversely, the MoCA demonstrates high specificity in our PCC population, functioning conservatively by showing strong agreement with the non-altered label. In addition, optimal MoCA cut-off scores vary as a function of age, education, language of administration, and race or ethnicity, with no universally accepted thresholds, and are associated with variability in sensitivity and specificity with wide confidence intervals across populations and clinical settings [8,9,10,11,12].

The MoCA combines multiple cognitive domains into a single composite score whereas ET measures reflect processes that may be sensitive to subtle inefficiencies in cognitive control. This pattern provides a clinical explanation for the complementary performance observed between ET and MoCA in the global composite analysis. While the improvement of the RF-ET + MoCA model over the standalone MoCA is modest in some domains, the confusion matrices showed that for the global composite, the integrated model enhances sensitivity relative to MoCA alone while improving specificity relative to the RF-ET model, integrating the strengths of both methods. Compared with the MoCA literature summarized by Islam et al. [11], the integrated RF-ET + MoCA model achieved sensitivity within the upper range reported for MCI populations, while maintaining moderate specificity, despite being applied to a more heterogeneous PCC cohort characterized by subtle cognitive impairment. This sensitivity in PCC supports the added value of multimodal integration for screening-level cognitive assessment. From a practical perspective, this performance improvement comes with minimal additional burden. ET tasks are brief, objective, and non-invasive, while the MoCA remains a widely accepted screening instrument with modest administration time. The combined approach offers a balance between diagnostic accuracy and clinical efficiency. Concretely, these results suggest that such a tool could potentially serve as a first-line screening filter in clinical settings. This approach may help identify patients who require more comprehensive neuropsychological evaluation but might otherwise be missed by the MoCA alone.

Several limitations should be considered. First, this study cross-sectional design offers only a snapshot of the relationships between cognitive performance and eye movements. As a result, these findings cannot show how cognitive symptoms evolve or recover over time, nor can they establish the prognostic value of ET measures for future cognitive outcomes Second, the MoCA performance is known to be influenced by demographic factors such as age and education introducing variability that may affect classification outcomes. Third, although ET provides distinct advantages, it exhibited lower specificity compared to multimodal approaches. Fourth, overall classification performance should be interpreted as moderate rather than definitive. Although the integrated ET and MoCA model improved discrimination relative to either modality alone, classification accuracy remains constrained by the subtle and heterogeneous nature of cognitive alterations in PCC. This heterogeneity may also be related to the frequent coexistence of fatigue, anxiety, and depression in PCC, conditions known to alter gaze behavior [54]. Consequently, some ET-derived alterations identified by the models may capture broader affective-attentional processes rather than purely cognitive impairment.

Finally, several methodological factors may limit generalizability and may introduce bias. The relatively small sample size and class imbalance may affect the performance estimates, particularly for outcomes with low prevalence of cognitive alteration. Class imbalance can influence model performance by biasing the classifier toward the majority class. To partially mitigate this, class imbalance was addressed during model training by including class weighting in the Random Forest hyperparameter search (see Table S3). When applied, class weighting increased the relative contribution of the underrepresented class, helping preserve sensitivity to minority cases. In addition, the transformation of continuous neuropsychological measures into binary labels may reduce information and introduce bias, especially near the selected threshold where small variations may result in different class assignments despite similar cognitive performance. To mitigate these issues, labels were derived from demographically adjusted z-scores and the composite outcomes across multiple domains.

Future longitudinal data will be required to determine whether ET-based markers are sensitive to change over time and whether they can accurately capture individual recovery trajectories and progression in the PCC population. Future studies with larger and more balanced cohorts will be necessary to further validate the robustness of ET-based classification and to refine decision thresholds across cognitive domains. Lastly, testing more cognitively demanding ET tasks may further increase the discriminative power of these models.

## 5. Conclusions

Cognitive impairment in PCC is often subjective, fluctuating, and varied across patients, making screening challenging. This study found that ET features provided process-level information that was useful when classifying global cognitive status, performance on individual tests or specific cognitive domains. While ET alone as an input to classification models showed lower discriminative performance than the established MoCA, combining both approaches into a RF model significantly improved the altered participant detection. This demonstrates that behavioral measures (ET) and performance-based tests (MoCA) complement each other effectively. This combined approach remained practical and interpretable for clinical use, supporting its potential as a scalable screening tool for cognitive impairment detection in PCC.

## Figures and Tables

**Table 1:** Descriptive statistics of neuropsychological test raw scores (*n* = 172). For each test, the table reports the minimum, maximum, mean and standard deviation.

Test	Min–Max	Mean Raw Score ± Standard Deviation
MoCA	16–30	26.14 ± 2.67
Digit span forward	3–9	5.52 ± 1.21
Digit span backward	2–8	4.35 ± 1.15
Digit symbol test	17–102	63.44 ± 16.07
TMT A	10–180	39.61 ± 19.62
TMT B	10–360	87.07 ± 46.09
SCWT word	12–144	86.51 ± 24.61
SCWT color	7–94	59.23 ± 15.12
SCWT color-word	4–96	36.02 ± 12.16
Phonological fluency	10–72	39.90 ± 12.01
Semantic fluency	8–38	20.23 ± 5.73

**Table 2:** Coefficient of determination (R^2^) and Root Mean Squared Percentage Error (RMSPE) using five regression algorithms (OLS, SGD, XGBoost, Random Forest, neural network) to predict each neuropsychological test raw score from ET features and age.

Test	OLS		SGD		XGBoost		Random Forest		Neural Network	
	R^2^	RMSPE	R^2^	RMSPE	R^2^	RMSPE	R^2^	RMSPE	R^2^	RMSPE
MoCA	−0.60	12.01	−0.19	12.43	−0.04	11.95	−0.04	11.97	−3.94	23.14
Digit span forward	−0.10	24.39	0.01	23.65	0.00	24.29	0.00	24.17	−0.13	23.50
Digit span backward	−1.25	54.29	−0.69	44.53	−0.02	32.44	−0.01	32.23	−0.52	32.69
Digit symbol test	−1.17	41.66	−0.40	39.69	0.01	37.74	0.01	37.52	−1.72	50.71
TMT A	−0.25	69.89	−0.59	71.23	−0.03	54.92	−0.02	55.76	−0.07	47.88
TMT B	−0.37	105.16	−0.01	102.27	0.04	102.71	0.05	103.06	−0.08	85.27
SCWT word	−0.14	73.79	−0.01	73.71	−0.03	74.45	−0.02	74.71	−1.37	89.48
SCWT color	−0.12	72.20	−0.35	72.09	−0.02	67.48	−0.02	68.76	−1.60	87.27
SCWT color-word	−0.08	80.37	−0.01	80.52	0.01	81.24	0.01	82.01	−1.08	105.09
Phonological fluency	−0.04	45.35	−1.09	54.76	0.01	49.82	0.00	50.22	−1.35	58.04
Semantic fluency	−0.35	36.11	−0.02	36.09	0.05	35.72	0.04	35.81	−0.75	41.02

**Table 3:** LDA, RF, standalone MoCA and RF-ET + MoCA classification performance for each neuropsychological test and cognitive composite. For each outcome the tables present the ROC-AUC and the percentage of participants classified as altered based on the z-score threshold (1 standard deviation) and raw score < 26 for the MoCA test. The highest AUC value for each outcome is shown in bold.

		ROC-AUC			
Test	% Altered Participants	LDA-ET	RF-ET	MoCA	RF-ET + MoCA
MoCA	38.37	0.48	0.50	**1.00**	**1.00**
Digit span forward	24.42	0.55	0.53	**0.75**	0.73
Digit span backward	25.58	0.62	0.63	**0.75**	0.73
Digit symbol	38.95	0.63	0.63	0.70	**0.79**
TMT-A	6.98	0.59	0.71	0.43	**0.74**
TMT-B	9.30	0.54	**0.72**	0.47	0.71
SCWT Word	36.63	0.52	0.53	**0.67**	0.61
SCWT Color	46.51	0.62	0.59	**0.73**	0.70
SCWT Color-Word	47.09	0.60	0.67	0.77	**0.80**
Phonological fluency	39.54	0.57	0.58	0.66	**0.69**
Semantic fluency	35.46	0.64	**0.65**	0.58	0.64
Attention	30.23	0.59	0.59	0.69	**0.74**
Executive function 1	66.28	0.64	0.62	0.75	**0.79**
Executive function 2	68.60	0.66	0.69	0.75	**0.81**
Processing speed 1	65.11	0.53	0.57	**0.72**	0.65
Processing speed 2	61.63	0.57	0.71	**0.72**	**0.72**
Global	63.65	0.70	0.68	0.73	**0.80**

## Data Availability

The dataset generated during this study has been deposited in the CORA-RDR repository. It will be made publicly available upon acceptance and publication of the article under a CC BY-NC-SA 4.0 license in CORA database.

## Supplementary Materials

The following supporting information can be downloaded at: https://www.mdpi.com/article/10.3390/jemr19030057/s1. Figure S1: Precision–Recall (PR) curves for the global cognitive composite classification models; Figure S2: Comparative performance metrics for the global cognitive composite classification models; Table S1: Metrics used for the characterization of fixation, smooth pursuit and pupil response; Table S2: Hyperparameter tunning of the regression modelling; Table S3: Optimal hyperparameters for each model and neuropsychological outcome.

## Abbreviations

The following abbreviations are used in this manuscript:

PCC	Post-COVID-19 Condition
ET	Eye-Tracking
MoCA	Montreal Cognitive Assessment
MCI	Mild Cognitive Impairment
LDA	Linear Discriminant Analysis
RF	Random Forest
OLS	Ordinary Least Squares
SGD	Stochastic Gradient Descent
XGB	Extreme Gradient Boosting

## References

[1] Soriano J.B., Murthy S., Marshall J.C., Relan P., Diaz J.V. (2022). A Clinical Case Definition of Post-COVID-19 Condition by a Delphi Consensus. Lancet Infect. Dis..

[2] Chen C., Haupert S.R., Zimmermann L., Shi X., Fritsche L.G., Mukherjee B. (2022). Global Prevalence of Post-Coronavirus Disease 2019 (COVID-19) Condition or Long COVID: A Meta-Analysis and Systematic Review. J. Infect. Dis..

[3] Lopez-Leon S., Wegman-Ostrosky T., Perelman C., Sepulveda R., Rebolledo P.A., Cuapio A., Villapol S. (2021). More than 50 Long-Term Effects of COVID-19: A Systematic Review and Meta-Analysis. Sci. Rep..

[4] Ariza M., Cano N., Segura B., Adan A., Bargalló N., Caldú X., Campabadal A., Jurado M.A., Mataró M., Pueyo R. (2022). Neuropsychological Impairment in Post-COVID Condition Individuals with and without Cognitive Complaints. Front. Aging Neurosci..

[5] Almeria M., Cejudo J.C., Sotoca J., Deus J., Krupinski J. (2020). Cognitive Profile Following COVID-19 Infection: Clinical Predictors Leading to Neuropsychological Impairment. Brain Behav. Immun. Health.

[6] García-Sánchez C., Calabria M., Grunden N., Pons C., Arroyo J.A., Gómez-Anson B., Lleó A., Alcolea D., Belvís R., Morollón N. (2022). Neuropsychological Deficits in Patients with Cognitive Complaints after COVID-19. Brain Behav..

[7] Ariza M., Cano N., Segura B., Adan A., Bargalló N., Caldú X., Campabadal A., Jurado M.A., Mataró M., Pueyo R. (2023). COVID-19 Severity Is Related to Poor Executive Function in People with Post-COVID Conditions. J. Neurol..

[8] Nasreddine Z.S., Phillips N.A., Bédirian V., Charbonneau S., Whitehead V., Collin I., Cummings J.L., Chertkow H. (2005). The Montreal Cognitive Assessment, MoCA: A Brief Screening Tool for Mild Cognitive Impairment. J. Am. Geriatr. Soc..

[9] Freitas S., Simões M.R., Alves L., Santana I. (2013). Montreal Cognitive Assessment: Validation Study for Mild Cognitive Impairment and Alzheimer Disease. Alzheimer Dis. Assoc. Disord..

[10] O’Driscoll C., Shaikh M. (2017). Cross-Cultural Applicability of the Montreal Cognitive Assessment (MoCA): A Systematic Review. J. Alzheimer’s Dis..

[11] Islam N., Hashem R., Gad M., Brown A., Levis B., Renoux C., Thombs B.D., McInnes M.D. (2023). Accuracy of the Montreal Cognitive Assessment Tool for Detecting Mild Cognitive Impairment: A Systematic Review and Meta-analysis. Alzheimer’s Dement..

[12] Tsai J.-C., Chen C.-W., Chu H., Yang H.-L., Chung M.-H., Liao Y.-M., Chou K.-R. (2016). Comparing the Sensitivity, Specificity, and Predictive Values of the Montreal Cognitive Assessment and Mini-Mental State Examination When Screening People for Mild Cognitive Impairment and Dementia in Chinese Population. Arch. Psychiatr. Nurs..

[13] Cena C.G., Costa M.C., Pazmiño R.S., Santos C.P., Gómez-Andrés D., Benito-León J. (2022). Eye Movement Alterations in Post-COVID-19 Condition: A Proof-of-Concept Study. Sensors.

[14] Larrazabal A.J., García Cena C.E., Martínez C.E. (2019). Video-Oculography Eye Tracking towards Clinical Applications: A Review. Comput. Biol. Med..

[15] Tao L., Wang Q., Liu D., Wang J., Zhu Z., Feng L. (2020). Eye Tracking Metrics to Screen and Assess Cognitive Impairment in Patients with Neurological Disorders. Neurol. Sci..

[16] Beltrán J., García-Vázquez M.S., Benois-Pineau J., Gutierrez-Robledo L.M., Dartigues J.-F. (2018). Computational Techniques for Eye Movements Analysis towards Supporting Early Diagnosis of Alzheimer’s Disease: A Review. Comput. Math. Methods Med..

[17] Opwonya J., Doan D.N.T., Kim S.G., Kim J.I., Ku B., Kim S., Park S., Kim J.U. (2022). Saccadic Eye Movement in Mild Cognitive Impairment and Alzheimer’s Disease: A Systematic Review and Meta-Analysis. Neuropsychol. Rev..

[18] Leigh R.J., Zee D.S. (2015). The Neurology of Eye Movements.

[19] Hodgson T.L., Ezard G., Hermens F., Hodgson T. (2019). Eye Movements in Neuropsychological Tasks. Processes of Visuospatial Attention and Working Memory.

[20] Pavisic I.M., Firth N.C., Parsons S., Rego D.M., Shakespeare T.J., Yong K.X.X., Slattery C.F., Paterson R.W., Foulkes A.J.M., Macpherson K. (2017). Eyetracking Metrics in Young Onset Alzheimer’s Disease: A Window into Cognitive Visual Functions. Front. Neurol..

[21] Bylsma F.W., Rasmusson D.X., Rebok G.W., Keyl P.M., Tune L., Brandt J. (1995). Changes in Visual Fixation and Saccadic Eye Movements in Alzheimer’s Disease. Int. J. Psychophysiol..

[22] Chan F., Armstrong I.T., Pari G., Riopelle R.J., Munoz D.P. (2005). Deficits in Saccadic Eye-Movement Control in Parkinson’s Disease. Neuropsychologia.

[23] Prasad S., Galetta S. (2010). Eye Movement Abnormalities in Multiple Sclerosis. Neurol. Clin..

[24] Gråwe R.W., Levander S. (1995). Smooth Pursuit Eye Movements and Neuropsychological Impairments in Schizophrenia. Acta. Psychiatr. Scand..

[25] Vinuela-Navarro V., Goset J., Aldaba M., Mestre C., Gay C., Cano N., Ariza M., Delàs B., Garolera M., Vilaseca M. (2023). Eye Movements in Patients with Post-COVID Condition. Biomed. Opt. Express.

[26] Opwonya J., Wang C., Jang K.-M., Lee K., Kim J.I., Kim J.U. (2022). Inhibitory Control of Saccadic Eye Movements and Cognitive Impairment in Mild Cognitive Impairment. Front. Aging Neurosci..

[27] Heuer H.W., Mirsky J.B., Kong E.L., Dickerson B.C., Miller B.L., Kramer J.H., Boxer A.L. (2013). Antisaccade Task Reflects Cortical Involvement in Mild Cognitive Impairment. Neurology.

[28] Fletcher W.A., Sharpe J.A. (1986). Saccadic Eye Movement Dysfunction in Alzheimer’s Disease. Ann. Neurol..

[29] Benito-León J., Lapeña J., García-Vasco L., Cuevas C., Viloria-Porto J., Calvo-Córdoba A., Arrieta-Ortubay E., Ruiz-Ruigómez M., Sánchez-Sánchez C., García-Cena C. (2024). Exploring Cognitive Dysfunction in Long COVID Patients: Eye Movement Abnormalities and Frontal-Subcortical Circuits Implications via Eye-Tracking and Machine Learning. Am. J. Med..

[30] Yang M., Cai C., Hu B. (2023). Clustering Based on Eye Tracking Data for Depression Recognition. IEEE Trans. Cogn. Dev. Syst..

[31] Knauer T.S., Mardin C.Y., Rech J., Michelson G., Stog A., Zott J., Steußloff F., Güttes M., Sarmiento H., Ilgner M. (2025). Evaluation of Stereopsis Performance, Gaze Direction and Pupil Diameter in Post-COVID Syndrome Using Machine Learning. Biomedicines.

[32] Goset J., Ariza M., Mestre C., Vinuela-Navarro V., Pérez-Mañá L., Vilaseca M., Cano N., Delàs B., Garolera M., Aldaba M. (2026). Eye Tracking and Machine Learning to Assess Cognitive Impairment in Post-COVID-19 Patients. Sci. Rep..

[33] Wechsler D. (1999). WAIS III Escala de Inteligencia de Wechsler Para Adultos-III.

[34] Ralph M. (1958). Reitan Validity of the Trail Making Test as an Indicator of Organic Brain Damage. Percept. Mot. Ski..

[35] Bowie C.R., Harvey P.D. (2006). Administration and Interpretation of the Trail Making Test. Nat. Protoc..

[36] Tombaugh T. (2004). Trail Making Test A and B: Normative Data Stratified by Age and Education. Arch. Clin. Neuropsychol..

[37] Golden C.J. (2005). Test de Colores y Palabras (Stroop).

[38] Benton A.L., Hamsher K. (1989). Multilingual Aphasia Examination.

[39] Pena-Casanova J., Quinones-Ubeda S., Gramunt-Fombuena N., Quintana-Aparicio M., Aguilar M., Badenes D., Cerulla N., Molinuevo J.L., Ruiz E., Robles A. (2009). Spanish Multicenter Normative Studies (NEURONORMA Project): Norms for Verbal Fluency Tests. Arch. Clin. Neuropsychol..

[40] Brainard D.H. (1997). The Psychophysics Toolbox. Spat. Vis..

[41] Pelli D.G. (1997). The VideoToolbox Software for Visual Psychophysics: Transforming Numbers into Movies. Spat. Vis..

[42] Nyström M., Holmqvist K. (2010). An Adaptive Algorithm for Fixation, Saccade, and Glissade Detection in Eyetracking Data. Behav. Res. Methods.

[43] Pedregosa F., Varoquaux G., Gramfort A., Michel V., Thirion B., Grisel O., Blondel M., Prettenhofer P., Weiss R., Dubourg V. (2011). Scikit-Learn: Machine Learning in Python. J. Mach. Learn. Res..

[44] Chollet F. Keras 2015. https://keras.io.

[45] Akiba T., Sano S., Yanase T., Ohta T., Koyama M. (2019). Optuna: A Next-Generation Hyperparameter Optimization Framework. Proceedings of the Proceedings of the 25th ACM SIGKDD International Conference on Knowledge Discovery & Data Mining.

[46] Knopman D.S., Pike J.R., Gottesman R.F., Sharrett A.R., Windham B.G., Mosley T.H., Sullivan K., Albert M.S., Walker K.A., Yasar S. (2024). Patterns of Cognitive Domain Abnormalities Enhance Discrimination of Dementia Risk Prediction: The ARIC Study. Alzheimer’s Dement..

[47] Petersen R.C. (2004). Mild Cognitive Impairment as a Diagnostic Entity. J. Intern. Med..

[48] Maudes J., Rodríguez J.J., García-Osorio C., García-Pedrajas N. (2012). Random Feature Weights for Decision Tree Ensemble Construction. Inf. Fusion.

[49] Lemos J., Eggenberger E. (2013). Saccadic Intrusions: Review and Update. Curr. Opin. Neurol..

[50] Majumdar D., Mondal K., Sahrawat T.R., Lima M.R.D. (2022). Eye Movement Metrics as Indicator of Cognitive Loading: A Systematic Review. Research Aspects in Biological Science.

[51] Gran Ekstrand A.C., Nilsson Benfatto M., Öqvist Seimyr G. (2021). Screening for Reading Difficulties: Comparing Eye Tracking Outcomes to Neuropsychological Assessments. Front. Educ..

[52] Kim M., Lee J., Lee S.Y., Ha M., Park I., Jang J., Jang M., Park S., Kwon J.S. (2024). Development of an Eye-Tracking System Based on a Deep Learning Model to Assess Executive Function in Patients with Mental Illnesses. Sci. Rep..

[53] Rizzo A., Ermini S., Zanca D., Bernabini D., Rossi A. (2022). A Machine Learning Approach for Detecting Cognitive Interference Based on Eye-Tracking Data. Front. Hum. Neurosci..

[54] Armstrong T., Olatunji B.O. (2012). Eye Tracking of Attention in the Affective Disorders: A Meta-Analytic Review and Synthesis. Clin. Psychol. Rev..

