# Supplementary material for: Machine Learning Integration of Eye-Tracking and Cognitive Screening for Detecting Cognitive Impairment
Source: J Eye Mov Res. 2026 May 20;19(3):57. doi: 10.3390/jemr19030057 (PMC13214842; doi:10.3390/jemr19030057)
Supplement: Supplementary file 1 [file jemr-19-00057-s001.zip › Supplementary Figure 1.pdf]

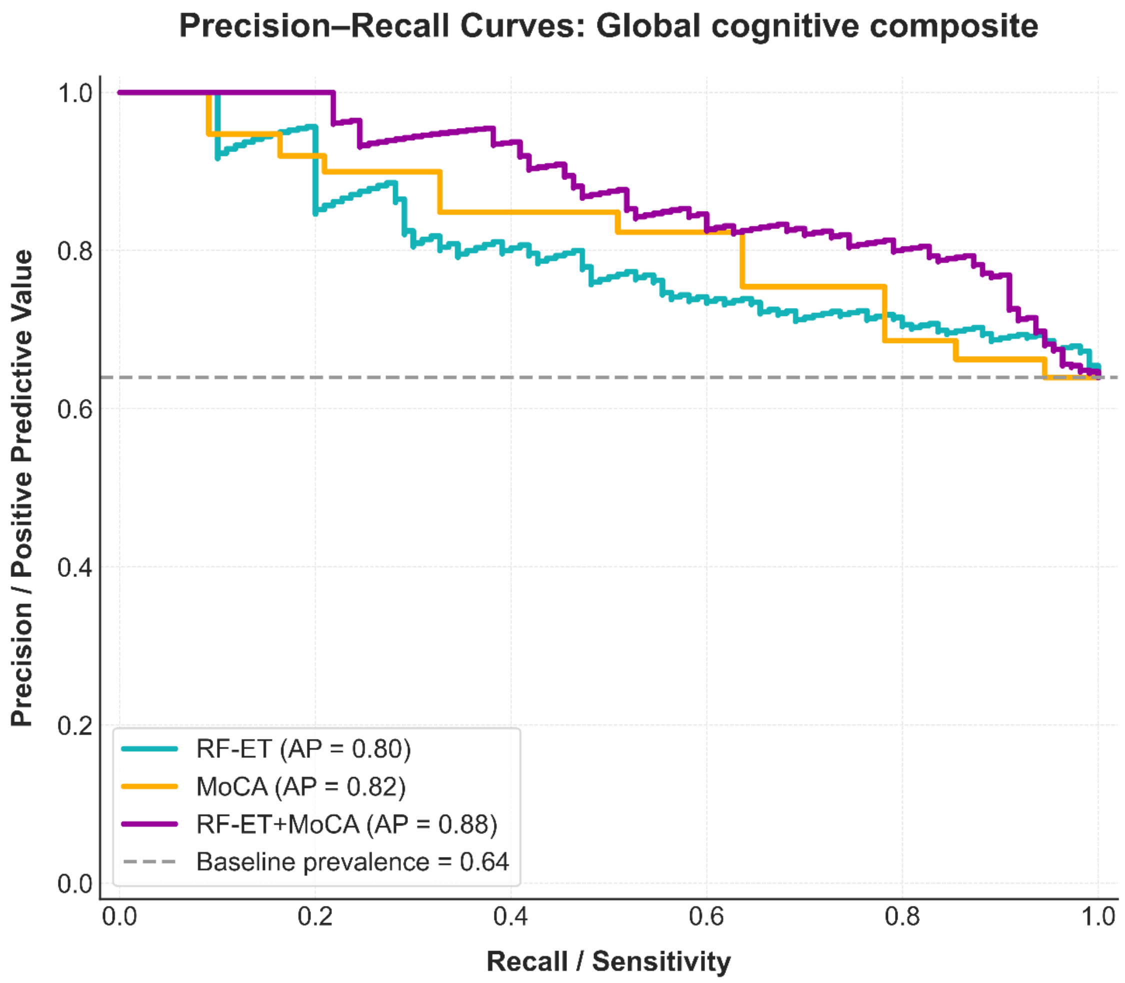

Supplementary Figure S1. Precision–Recall (PR) curves for the global cognitive composite classification models. The multimodal RF-ET+MoCA model (purple) achieved the highest Average Precision (AP = 0.88), outperforming both the RF-ET model (teal; AP = 0.80) and the MoCA-only classifier (orange; AP = 0.82). The dashed grey line represents the baseline prevalence of altered cognitive performance in the sample (0.64).
