# Supplementary material for: Machine Learning Integration of Eye-Tracking and Cognitive Screening for Detecting Cognitive Impairment
Source: J Eye Mov Res. 2026 May 20;19(3):57. doi: 10.3390/jemr19030057 (PMC13214842; doi:10.3390/jemr19030057)
Supplement: Supplementary file 1 [file jemr-19-00057-s001.zip › Supplementary Figure 2.pdf]

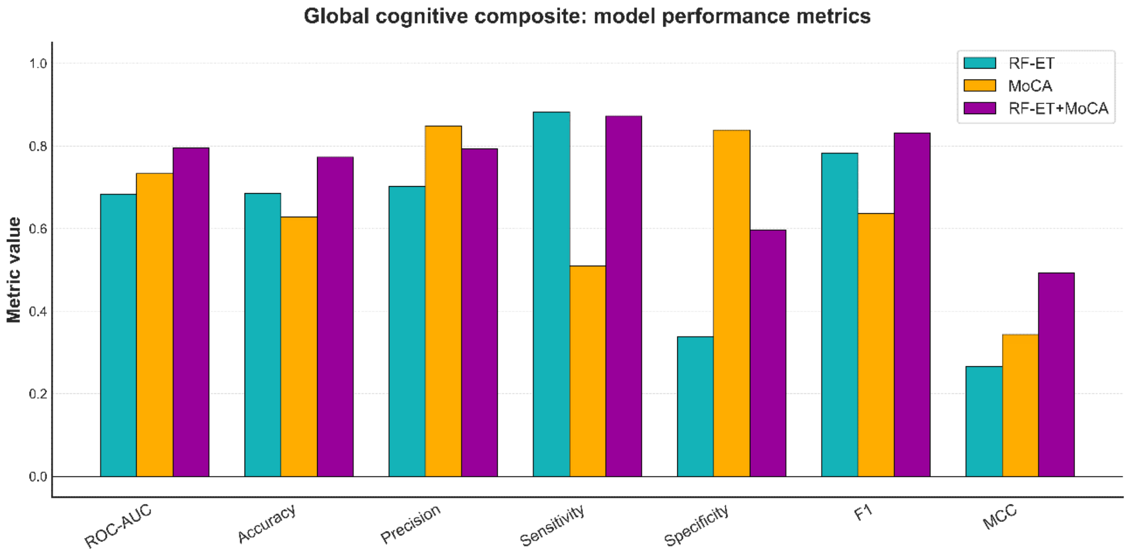

Supplementary Figure S2. Comparative performance metrics for the global cognitive composite classification models. The multimodal RF-ET+MoCA classifier (purple) demonstrated the best overall performance across most evaluation metrics, including ROC-AUC, accuracy, F1-score, and Matthews Correlation Coefficient (MCC), compared with the RF-ET (teal) and MoCA-only (orange) models. While the MoCA classifier achieved the highest specificity and precision, the multimodal approach provided a more balanced trade-off between sensitivity and specificity, resulting in superior global classification performance.
