# Supplementary material for: Machine Learning Integration of Eye-Tracking and Cognitive Screening for Detecting Cognitive Impairment
Source: J Eye Mov Res. 2026 May 20;19(3):57. doi: 10.3390/jemr19030057 (PMC13214842; doi:10.3390/jemr19030057)
Supplement: Supplementary file 1 [file jemr-19-00057-s001.zip › Table S1.pdf]

Table S1. Metrics used for the characterization of fixation, smooth pursuit and pupil response.

	**Definition**
**Fixation**	
All metrics are calculated for the two paradigms: before distractors appear and with distractors	
RMS [º]	Root Mean Square error representing the variability in gaze position computed as the distance between measurements recorded by the eye tracker and the central stimulus. This parameter reflects the precision and accuracy of fixation, with lower RMS values indicating greater stability and less fluctuation in gaze.
Nº of microsaccades	Number of saccades during the fixation task with an amplitude < 1º.
**Smooth pursuit**	
All metrics are calculated for the three trajectories: sinusoidal, horizontal and vertical	
RMS [º]	Root Mean Square error denoting the distance between the target and gaze positions. For each gaze position, the RMS is computed to the position of the target at the same timestamp, with lower RMS values indicating greater tracking accuracy.
Gain	Ratio of eye velocity to stimulus velocity.
Saccadic component [%]	Percentage of time spent on saccades during the smooth pursuit task.
**Pupil response**	
Miosis time [ms]	Time that elapses between the LED is switched on and the maximum constriction is achieved.
Area reduction [%]	Area reduction (%) during miosis. The initial value is obtained as the mean pupil area within the last second before switching on the LED and the final value corresponds to the maximum constriction measured.
Area enlargement [%]	Area growth (%) during mydriasis. The initial value is obtained as that corresponding to maximum constriction, and the final one as the mean pupil area within the last second of the testing condition (75 s).
