# Supplementary material for: Machine Learning Integration of Eye-Tracking and Cognitive Screening for Detecting Cognitive Impairment
Source: J Eye Mov Res. 2026 May 20;19(3):57. doi: 10.3390/jemr19030057 (PMC13214842; doi:10.3390/jemr19030057)
Supplement: Supplementary file 1 [file jemr-19-00057-s001.zip › Table S2.pdf]

Table S2. Supplementary table, hyperparameter tunning of the regression modelling

Model	Key hyperparameters
**SGD**	loss = squared_error; penalty = elasticnet; α = 0.00965; l1_ratio = 0.987
**XGBoost**	n_estimators = 116; learning_rate = 0.0143; max_depth = 2; subsample = 0.709; colsample_bytree = 0.786; reg_alpha = 0.0783; reg_lambda = 9.95; min_child_weight = 7.38
**Random Forest**	n_estimators = 580; max_depth = 2; min_samples_split = 7; min_samples_leaf = 10; max_features = sqrt; bootstrap = TRUE
