# Supplementary material for: Machine Learning Integration of Eye-Tracking and Cognitive Screening for Detecting Cognitive Impairment
Source: J Eye Mov Res. 2026 May 20;19(3):57. doi: 10.3390/jemr19030057 (PMC13214842; doi:10.3390/jemr19030057)
Supplement: Supplementary file 1 [file jemr-19-00057-s001.zip › Table S3.pdf]

Table S3. Optimal hyperparameters for each model and neuropsychological outcome. Hyperparameters were independently optimized for each outcome using Bayesian optimization (Optuna) to maximize ROC-AUC. Separate configurations are reported for Linear Discriminant Analysis (LDA) and Random Forest (RF) models

Test	Model	solver	shrinkage	n_estimators	max_depth	min_samples_split	min_samples_leaf	max_features	class_weight	bootstrap
MoCA	LDA	eigen	1							
	RF			172	2	2	9	log2	balanced_subsample	1
Digit span forward	LDA	eigen								
	RF			105	2	15	4	log2	balanced	1
Digit span backward	LDA	lsqr	0.15							
	RF			198	5	3	10		balanced	0
Digit symbol test	LDA	lsqr								
	RF			791	2	6	7	sqrt		1
TMT A	LDA	eigen	0.01							
	RF			301	3	17	3	log2		0
TMT B	LDA	lsqr								
	RF			661	12	10	1			1
SCWT word	LDA	eigen								
	RF			726	3	19	1	sqrt		1
SCWT color	LDA	lsqr								
	RF			349	2	4	5	log2		1
SCWT color-word	LDA	lsqr								
	RF			226	6	7	3	sqrt		1
Phonological fluency	LDA	svd								
	RF			113	2	11	4	sqrt	balanced	1
Semantic fluency	LDA	svd								
	RF			697	7	7	9	log2		0
Attention	LDA	lsqr	0.45							
	RF			103	7	12	1		balanced	1
Executive function 1	LDA	lsqr	1							
	RF			169	7	9	4		balanced_subsample	1
Executive function 2	LDA	lsqr	1							
	RF			107	11	4	9		balanced_subsample	0
Processing speed 1	LDA	lsqr								
	RF			144	3	7	10		balanced	0
Processing speed 2	LDA	lsqr	1							
	RF			648	2	14	5	log2	balanced_subsample	1
Global	LDA	eigen	0.37							
	RF			176	6	3	2	sqrt	balanced	1

.
